# The Feasibility of Using the National PulsePoint Cardiopulmonary Resuscitation Responder Network to Facilitate Overdose Education and Naloxone Distribution: Protocol for a Randomized Controlled Trial

**DOI:** 10.2196/57280

**Published:** 2024-03-29

**Authors:** Jon Agley, Cris Henderson, Dong-Chul Seo, Maria Parker, Lilian Golzarri-Arroyo, Stephanie Dickinson, David Tidd

**Affiliations:** 1 Prevention Insights Department of Applied Health Science, School of Public Health Bloomington Indiana University Bloomington Bloomington, IN United States; 2 Department of Applied Health Science School of Public Health Bloomington Indiana University Bloomington Bloomington, IN United States; 3 Department of Epidemiology and Biostatistics School of Public Health Bloomington Indiana University Bloomington Bloomington, IN United States; 4 Biostatistics Consulting Center School of Public Health Bloomington Indiana University Bloomington Bloomington, IN United States

**Keywords:** naloxone, PulsePoint, randomized controlled trial, RCT, first responder, overdose, community engagement, citizen mobilization, opioids, Narcan, mobile phone

## Abstract

**Background:**

The use of naloxone, an opioid antagonist, is a critical component of the US response to fatal opioid-involved overdoses. The importance and utility of naloxone in preventing fatal overdoses have been widely declaimed by medical associations and government officials and are supported by strong research evidence. Still, there are gaps in the current US national strategy because many opioid-involved overdose fatalities have no evidence of naloxone administration. Improving the likelihood that naloxone will be used to prevent fatal overdoses is predicated on facilitating an environment wherein naloxone is available near each overdose and can be accessed by someone who is willing and able to use it. How to accomplish this on a national scale has been unclear. However, there exists a national network of >1 million cardiopulmonary resuscitation (CPR) layperson responders and 4800 emergency responder agencies linked through a mobile phone app called *PulsePoint Respond*. *PulsePoint* responders certify that they are trained to administer CPR and are willing to respond to possible cardiac events in public. When such an event occurs near their mobile phone’s location, they receive an alert to respond. These motivated citizens are ideally positioned to carry naloxone and reverse overdoses that occur in public.

**Objective:**

This randomized controlled trial will examine the feasibility of recruiting first responder agencies and layperson CPR responders who already use *PulsePoint* to obtain overdose education and carry naloxone.

**Methods:**

This will be a 3-arm parallel-group randomized controlled trial. We will randomly select 180 first responder agencies from the population of agencies contracting with the PulsePoint Foundation. The 3 study arms will include a standard recruitment arm, a misperception-correction recruitment arm, and a control arm (1:1:1 allocation, with random allocation stratified by zip code designation [rural or nonrural]). We will study agency recruitment and, among the agencies we successfully recruit, responder certification of receiving overdose and naloxone education, carrying naloxone, or both. Hypothesis 1 contrasts agency recruitment success between arms 1 and 2, and hypothesis 2 contrasts the ratios of layperson certification across all 3 arms. The primary analyses will be a logistic regression comparing the recruitment rates among the arms, adjusting for rural or nonrural zip code designation.

**Results:**

This study was reviewed by the Indiana University Institutional Review Board (20218 and 20219). This project was funded beginning September 14, 2023, by the National Institute on Drug Abuse.

**Conclusions:**

The hypotheses in this study will test whether a specific type of messaging is particularly effective in recruiting agencies and layperson responders. Although we hypothesize that arm 2 will outperform the other arms, our intention is to use the best-performing approach in the next phase of this study if any of our approaches demonstrates feasibility.

**Trial Registration:**

OSF Registries osf.io/egn3z; https://osf.io/egn3z

**International Registered Report Identifier (IRRID):**

PRR1-10.2196/57280

## Introduction

### Background

In 2017, the US Acting Secretary of Health and Human Services formally declared that the opioid overdose crisis in the United States is a public health emergency [1]. Since that time, the prevalence of morbidity and mortality related to opioid overdoses has gotten substantively worse [2]. Beyond the prima facie impact of tens of thousands of overdose-involved deaths annually, the secondary impacts of the crisis strain the health care system across numerous axes; for example, even in 2013, when annual fatal prescription overdose incidence was “only” 16,235 persons, scientists at the Centers for Disease Control and Prevention found that these fatal cases accrued US $21.5 billion in costs [3]. The magnitude of the emergency is such that even in the midst of major global news on multiple fronts, news stories about fatal opioid overdoses are ubiquitous, often with calls from family members to build awareness and seek solutions [4]. Overdose and harm reduction strategies (including naloxone administration) have also received direct attention from the federal government, including as part of the 2023 State of the Union address [5].

### Naloxone Is a Critical Part of the US Overdose Emergency Response

Naloxone (including Narcan, Kloxxado, Evzio, Zimhi, and other formulations) is an opioid antagonist that can be used to reverse an opioid overdose and restore breathing, converting a potentially fatal overdose into a nonfatal overdose [6,7]. Even before the formal declaration of a public health emergency around opioids, the Department of Health and Human Services included expanded use and distribution of naloxone in its 3 priority areas to address the overdose epidemic [8]. The importance and utility of naloxone have been widely declaimed, including by the American Medical Association (“should be available almost everywhere”) [9], the American Society of Addiction Medicine (“remarkably effective, inexpensive and safe”) [10], and the US Surgeon General (“Be Prepared. Get Naloxone. Save a Life.”) [11]. Such statements are predicated on strong evidence of, and arguments for, naloxone’s effectiveness and utility in reducing fatal opioid-involved overdoses [12-16].

### Barriers Exist That Inhibit Proliferation of Expedient and Effective Overdose Responses

Data from 2019 indicate that in the majority of fatal opioid-involved overdoses, there is no evidence of naloxone administration [17]. Given the current evidence for the effectiveness of overdose education and naloxone distribution (OEND) programs [12-16], the national expansion of such programs [18,19], and simultaneous high rates of fatal opioid-involved overdoses without naloxone administration [17], we might reasonably conclude that there are one or more gaps in the current US national approach to opioid-involved overdose reversal.

For many years, the lack of availability of naloxone has been seen as a primary barrier. Most states have passed legislation intended to increase layperson access to naloxone and provide limited legal immunity to those who administer naloxone to persons experiencing an opioid-related overdose [10]. By 2021, a total of 47 states and Washington, District of Columbia, had enacted both Good Samaritan and naloxone access laws [20], and programs to facilitate distribution of naloxone to laypersons have steadily become more common [18], especially since 2010 [19]. Unfortunately, a 2018 study in Indiana and Arizona found that such policies did not always sufficiently facilitate layperson access [21]. The availability of naloxone *at cost* will likely increase in the United States in the next several years given a recent 19-0 US Food and Drug Administration advisory committee vote in favor of granting some naloxone products over-the-counter (OTC) status [22]. However, national data from a similar OTC change in Australia suggest that people may not automatically begin purchasing naloxone in response [23] (eg, other factors such as willingness may be salient).

More ubiquity in carrying and being willing to use naloxone is critical because, as described by Kim et al [24], the efficacy of naloxone is fundamentally time dependent. Brain injury from hypoxia is more likely the longer oxygen deprivation occurs [25]. In some cases, overdose death from heroin can be extremely rapid [26], and death typically occurs within 1 to 3 hours [27]. Overdoses from fentanyl and fentanyl analogs often present differently than other opioid overdoses and may be even more likely to rapidly result in fatality [28]. Thus, it is important that naloxone is *present at or near the scene* and *used as quickly as possible.* Unfortunately, despite a surge in OEND programs in the United States, relatively few people carry and are willing to use naloxone [29].

### Misperceptions About Naloxone and Overdose May Inhibit OEND Programs

Evidence suggests that misperceptions about overdose and naloxone, as well as stigmatizing beliefs about people who use drugs, may affect both layperson and first responder willingness and interest in carrying and using naloxone [29-33]; for example, first responders may be ambivalent about “providing naloxone to those they deem undeserving” [34], and a review found that stigmatizing attitudes among first responders may reduce the likelihood of bystander response [35]. Some first responders have noted that laypersons in their communities may fear “real and perceived cultural opposition” to harm reduction strategies [36]. Such reticence and stigma may even persist after OEND training [29,37] and have been described as fundamentally hindering the US response to the opioid overdose crisis [38].

To the best of our knowledge, the prevalence of specific belief statements about naloxone and overdose that do not align with current scientific evidence had not been explored in depth until 2022 [39]. We conducted a study (using the Prolific platform) consisting of 702 respondents who were representative of the US adult population across race, ethnicity, gender, and age. Several misperceptions around naloxone and overdose were fairly common in our sample, and there was a surprisingly large minority of participants (approximately one-seventh of the sample) who believed that users could “get high on naloxone” [39]. We also observed potential evidence of cognitive dissonance about OEND; specifically, large latent profiles simultaneously believed, in the framed context of naloxone reversing an opioid overdose, that “if trained and provided with naloxone, bystanders can effectively prevent overdoses in the community” and “opioid users will use more opioids if they know they have access to naloxone” [39].

### Our Proposed Study to Mitigate Barriers to Effective Overdose Response

Improving the likelihood that naloxone will be used to prevent fatal overdose is logically predicated on facilitating an environment wherein naloxone is available near each overdose and can be accessed by someone who is willing and able to use it. Conceptually, in a given community, the likelihood of an opioid overdose in the given community being reversed with naloxone is proportional to the likelihood that a trained person with access to naloxone is nearby, willing to administer it, and aware of the need. As noted, current approaches to OEND have not succeeded in reaching the threshold of sufficient coverage. With too few trained persons and sparse coverage, overdose survivability can be subject to the whims of chance, something implicitly recognized even in how lay news sometimes covers overdose reversals (“finds herself in the right place at the right time” [40]).

Recent, innovative pilot work demonstrated that a smartphone app (*UnityPhilly*) could support overdose reversal in a small group of volunteers [41]. Other innovative projects include multifaceted technology-based efforts by Brave Technology Co-Op [42]. In these cases, building to scale will take substantial time and investment. However, it may also be possible to integrate OEND within widespread but existing systems that perform other but similar services. Our particular interest is in a large, highly engaged system for cardiopulmonary resuscitation (CPR) that already exists nationally (*PulsePoint*), with >1,085,000 active monthly layperson users across approximately 4800 communities overseen by >755 agencies [43]. These individuals respond to unconscious and unresponsive persons within a prespecified radius of their smartphone based on alerts generated by local first responder agencies. While *PulsePoint* does not specifically generate alerts for an opioid overdose, the designation *unconscious and unresponsive* can refer to individuals possibly in need of CPR or those who may need overdose reversal; therefore, these laypersons are likely already responding to possible overdoses. However, they are neither systematically being encouraged to receive training around overdose and naloxone nor being reminded to carry naloxone.

We believe that the *PulsePoint* network may be an effective means of achieving widespread OEND coverage in the United States. With this preliminary study, our goal is to better understand whether and how it is feasible to recruit first responder organizations and *PulsePoint*-connected laypersons to broaden the scope of their voluntary service to include opioid overdose reversal.

### Objectives

#### Overview

We will conduct a feasibility trial to assess whether, in a random sample of 180 agencies where *PulsePoint* is already active, revised procedures (hereinafter *PulsePoint-OD*) to facilitate OEND can successfully recruit first responder agencies and layperson responders. For this study, *agency* refers to a single implementation site where *PulsePoint* is active and that is bound by the service area or jurisdiction of the first responder agency collaborating with *PulsePoint* (we provide more detailed information about agencies in the *Methods* section).

Using a parallel-group randomized controlled trial design, we will randomize 180 agencies using a 1:1:1 allocation ratio within strata to 1 of 3 arms: arm 1 (*PulsePoint-OD*, which will include the recruitment of coordinating community first responder agencies; targeted recruitment, eg, push messages, aimed toward 100% of the active *PulsePoint* users in these communities; ongoing solicitation for training of these users; and naloxone facilitation), arm 2 (same as arm 1 but with stigma-focused misinformation-debunking messages embedded), or arm 3 (a control condition).

#### Hypotheses

We will test the following hypotheses:

- H1: more first responder agencies will be successfully recruited by arm 2 than by arm 1.
- H2: more layperson responders will report engaging with OEND programming in arm 2 than in arms 1 or 3 and in arm 1 than in arm 3.

As an exploratory follow-up, we will conduct incentivized qualitative follow-up interviews with agencies to better understand barriers to participation and how to improve uptake feasibility.

## Methods

**Figure 1:**
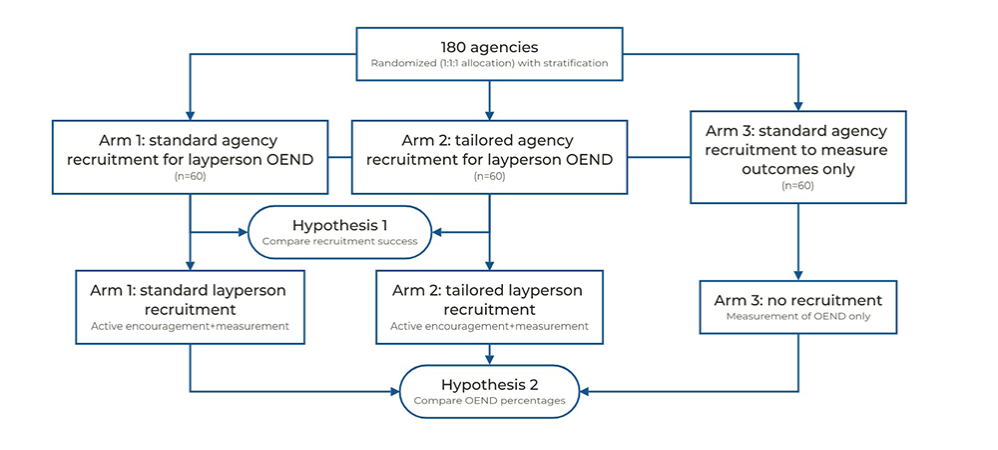
Conceptual diagram of the study and hypotheses. OEND: overdose education and naloxone distribution.

As the participant types for H1 and H2 are different, we provide separate methodologies for each hypothesis. A conceptual diagram of the study and hypotheses is provided in Figure 1.

### H1: First Responder Agencies

#### Parameters

The following parameters describe the interrelationships between communities and agencies for this study:

- The PulsePoint Foundation reports that approximately 4800 communities currently use the *PulsePoint* app.
- Community participation and the use of the *PulsePoint* app are supported by agency contract holders; there are >755 such contracts in place.
- A contract-holding agency can serve multiple communities (eg, a county 911 office can offer *PulsePoint* to 9 different communities in the county, such as townships). An agency can also serve a single community (eg, a city fire department can offer *PulsePoint* to that city only).
- Any number of *additional* first responder agencies (eg, fire or police departments, emergency medical services, 911 call centers, or related entities) can contribute to a community or cluster of communities covered by a single *PulsePoint* contract, but these agencies are not contract holders.

When *PulsePoint* is introduced to a community for layperson use, the PulsePoint Foundation neither directly recruits nor interfaces with individual lay responders. Instead, the contracted first responder agencies conduct local recruitment and have the ability to send *push messages* (app-driven notices and messages) to users. Therefore, to integrate OEND into the *PulsePoint* network (eg, *PulsePoint-OD*) and for our study team to directly contact the subset of active users within each study community, it is necessary that the contract-holding first responder agencies in each community agree to participate. This first phase of the overall study (and the first hypothesis) pertains to agency-level recruitment.

#### Participants, Eligibility, and Assignment

We will use the complete list of *PulsePoint* contracting agencies as constituted approximately 3 months before the study start date (to ensure sufficient experience with the *PulsePoint* program) as the population from which to draw a random sample. At present, this list includes >755 agencies, but the actual number will vary depending on the final start date and changes in *PulsePoint* membership.

All active agencies on the final list will be eligible for sampling and randomization, with 2 exceptions. First, an agency will be excluded (and a replacement resampled and assigned to the same study arm) if further communication identifies that it is not actually a *PulsePoint* subscriber. Second, we will exclude agencies a priori or a posteriori if we learn of a similar project being conducted, or having been conducted, with *PulsePoint* with the agency. Random sampling of communities will be accomplished by having a study statistician generate a random sequence of numbers, including all values between *1* and *X*, where *X* is the total number of eligible agencies. These numbers will be directly overlaid on the list of agencies, and agencies with the lowest 180 numbers will be selected. These numbers will also be carried forward to indicate the random arm assignment.

Agencies will be randomly assigned to a study arm using stratified allocation, with a 1:1:1 allocation ratio. Specifically, one-third of the rural agencies will be randomly assigned to each study arm, and separately, one-third of the nonrural agencies will be randomly assigned to each study arm. We will determine rural status using the definition from the US Office of Management and Budget (zip codes within metropolitan areas with an urban core of ≥50,000 people are not rural, and all other areas are rural [44]). In cases where agencies hold *PulsePoint* contracts that cover both rural and nonrural areas, we will assign a status based on the location of the numeric majority of the population in the area. Arm assignment will be according to sequential numbers (from the simple random sample; therefore, this allocation approach is also by definition random) within each stratum (eg, if there are 60 rural agencies, the 20 rural agencies with the lowest random numbers will be assigned to arm 1). This procedure will ensure allocation concealment from the rest of the investigative team until the moment of assignment. Agencies will not be informed that they have been assigned to a specific trial arm; they will simply be contacted using the procedures developed for that arm. Thus, this can be considered a blinded trial.

#### Establishing Correspondence With Local PulsePoint Decision Makers

Although the PulsePoint Foundation will provide us with a list of participating agencies, its database does not include raw contact details (eg, telephone number and email address), and even these details may not link our study team with the people within the agency who manage the *PulsePoint* system. On the basis of discussions with the PulsePoint Foundation, the following examples describe cases that have occurred in the past:

- The point of contact on the agency list may be the accounting or billing representative for the agency, not the person who manages the program, especially among long-term subscribers.
- The decision makers for the agency may be different now than when the subscription to *PulsePoint* was purchased.

We will need to locate contact information for agencies and verify that the contact details link us with the person who manages the *PulsePoint* subscription, and if not, identify who in the agency does so and how to contact them. In other words, while our trial is intended to study recruitment strategies, we must first ensure that we can access the appropriate individuals at an agency. Once we have identified these points of contact, we can begin recruitment at the agency.

#### Intervention (Recruitment) Arm 1

##### Overview

**Figure 2:**
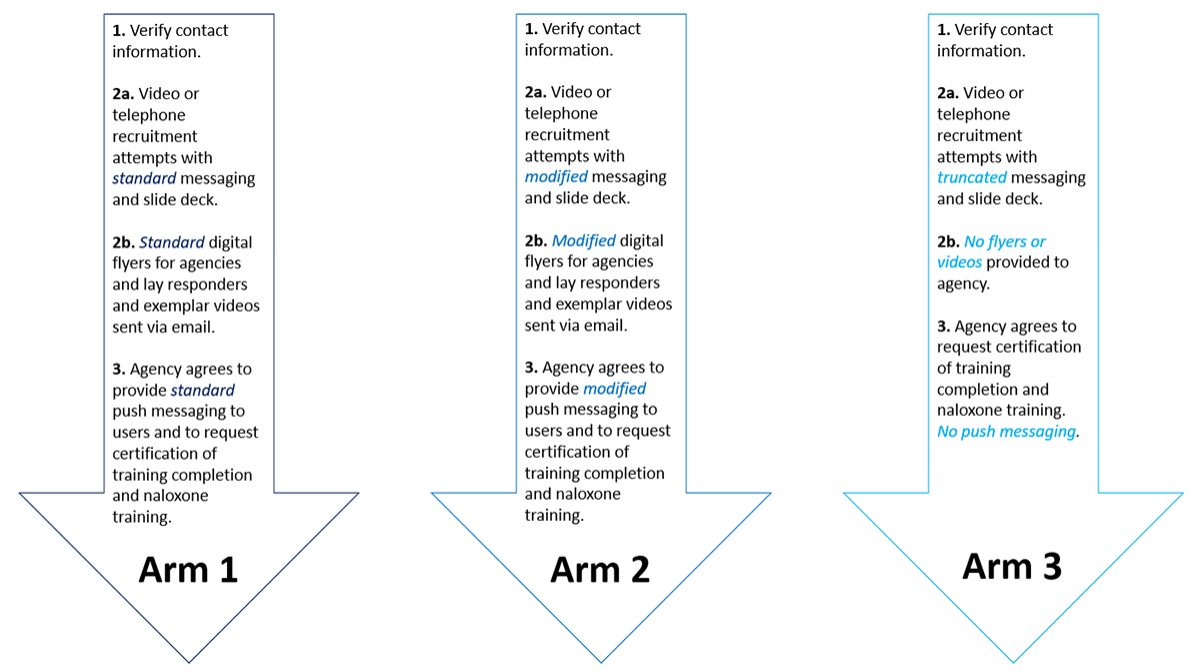
Differing agency recruitment procedures for the PulsePoint-OD study by study arm.

The minimum goal of agency recruitment is to reach a memorandum of understanding (MOU) with agencies in which they agree to encourage their layperson *PulsePoint* responders to attend OEND programming and to carry naloxone (arms 1 and 2) and to indicate whether they have done so (arms 1, 2, and 3; Figure 2). The agencies will be asked to accomplish this using push messages prepared by our team and sent out by the agencies; the specific nature of these agency activities is outlined in the *H2: Layperson Responders* section.

##### Development

Our agency recruitment materials will be developed during the first phase of the study and provided in full when the study results are published. Oversight and review of marketing development will be provided by members of the study team and marketing consultants external to the study team. Review of all marketing materials will be granted to the PulsePoint Foundation (a not-for-profit 501[c][3] organization) to ensure consistency with the organization’s branding. As a result of the development timeline, the exact phrases and examples used to illustrate components of the recruitment processes may change between the submission of this paper and the launch of the study.

##### Approaches

To be replicable at scale, recruitment will be conducted using a combination of video or telephone correspondence (preferably Zoom [Zoom Video Communications, Inc], but telephone where necessary), web links, and emails containing tangible materials. A full recruitment attempt with fidelity to our model will consist of providing emailed materials and web links and attempting between 6 and 10 telephone contacts over approximately 6 months, based on best practice telephone recommendations from the American Association for Public Opinion Research [45]. Recruitment efforts will stop in the event that (1) an agency agrees to participate in the project, (2) an agency asks not to be contacted about the project again, (3) the aforementioned recruitment approaches clearly seem unlikely to yield a change in disposition, or (4) the recruitment timeline ends without the agency making a decision.

##### Materials

Members of our project staff will develop a short visual presentation to be used as a guideline for videoconference recruitment, along with a parallel set of core written or verbal statements reflecting the content of the presentation in cases where only telephone calls are possible. Drawing lessons from our previous successful agency and organization recruitment experiences [46-49], both the presentation and written statements will cover a limited list of important concepts that need to be imparted (eg, “Participating in this project will require very little time from your agency – we are only asking you to send out one push message each month.”). While moving through these concepts, we will allow conversation to flow freely (eg, semistructured messaging) and also use live documentation of key concepts to ensure message fidelity.

The tangible documentation will consist of digital flyers based on the open-source marketing tools developed by the PulsePoint Foundation for general community agency recruitment, including the use of similar graphical assets and fonts. However, flyers will be differentiated sufficiently from the default *PulsePoint* flyers to make it clear that the organization itself is not conducting this project (eg, we will not use the *PulsePoint* logo). For reference, *PulsePoint*-branded recruitment flyers can be viewed by clicking on the hyperlink provided in the associated reference [50]. As community agencies have already committed to offering standard *PulsePoint* services, we expect that distributing these modified documents to agencies will be a high-leverage recruitment approach. Key messaging will be drawn from local demonstration projects by the study team (eg, “Be a Lifesaver” and “Citizen Opioid Responders”) [51] as well as general marketing and recruitment principles.

As supplemental recruitment content, exemplar videos will be drawn from work produced by the Dearborn County Health Department in Lawrenceburg, Indiana, in collaboration with the study authors to create awareness of this type of initiative and will be included as web links to YouTube versions of the videos [51].

##### After Recruitment

When communities agree to participate, they will be asked to sign an MOU regarding their participation and to respond to a small set of readiness and implementation items, such as whether the organization is already involved in naloxone distribution and whether there are local laws pertaining to opioid overdose reversal. This information will be used to locally tailor piped text into messages used for H2 (layperson recruitment).

#### Intervention (Recruitment) Arm 2

The recruitment procedures for arm 2 will be similar to those outlined for arm 1. The primary difference will be that the messaging in arm 2 will proactively address several misperceptions or stigmatizing ideas about overdose and naloxone that our recent research has suggested may be prevalent [39]. In particular, content in the fact sheets and scripts for recruitment used in arm 2 will address and preempt the following scientifically unsupported ideas (potentially among others):

- “Naloxone causes risk compensation or presents a moral hazard”: it is fairly well established that OEND is not associated with increased opioid use [52-55], nor is it associated with reduced risk perceptions for heroin use [56].
- “Repeated overdoses are inevitable and people who experience nonfatal overdoses will die of an overdose in the near future”: while individuals who nonfatally overdose are at meaningfully increased risk, especially if they do not matriculate into a cascade of care, the substantive majority will not overdose again in the near future, and even fewer will die of a fatal overdose in the near future [57-60].
- “Individuals can get high from naloxone”: while this is not possible [6], our study found that 14.2% of our nationally-representative sample still believed this claim [39] to some extent.

Though brief, our debunking content will follow specific procedures that recent research (particularly around COVID-19 misinformation and vaccine hesitancy) has found to improve the efficacy of debunking messaging. These include ensuring the credibility of the source of the factual correction [61] and including a sufficient level of detail and explanation for *why* the facts are correct and the misperceptions are not [62]. This level of detail will vary in extensiveness depending on the format of a given piece of communication. The use of such short-format corrections does not seem to result in a backfire effect [63]; for example, a statement made in the arm 1 recruitment flyer (standard) might be, “Overdoses involving opioids are a public health emergency in the United States (US). The US Surgeon General recommends: ‘Be Prepared. Get Naloxone. Save a Life.’” The permutation for the arm 2 recruitment fact sheet (modified), in parallel, would be, “Rapid use of naloxone (Narcan) nasal spray is often an overdose victim’s best chance for survival. Studies show that opioid use in a community does not increase when naloxone is widely available.”

#### Inert Arm for H1 (Arm 3)

Arm 3 recruitment will mirror recruitment for arm 1, except that the stated goal for the agencies will be only to ask layperson respondents to indicate at 3 different time points whether they have attended OEND programming or carry naloxone (eg, no encouragement or messaging campaign will occur in this arm). This arm will not be actively analyzed when we test H1.

#### Outcome Variables and Measurement

In H1, we hypothesize that more community first responder agencies will agree to participate in the *PulsePoint-OD* program when contacted using procedures in arm 2 (tailored messaging) compared to procedures in arm 1 (standard messaging) within 6 and 12 months of initial contact. Our primary outcome to test this hypothesis will be the number of agencies that agree to participate in the project as a percentage of the number of agencies with which we have been able to identify appropriate points of contact for recruitment. This metric *M* will be computed as follows:

- Each agency from the initial PulsePoint Foundation list that is randomized to participate in the study will be given a value *A* that is set at *0*, which indicates that we have not yet identified the appropriate point of contact at the agency.
- When we have identified a point of contact who can discuss *PulsePoint*, we will set the value for *A* at *1*.
- To define when a community first responder agency has agreed to participate, each agency will be assigned an ordinal value *R* ranging from *0* to *2*, where *0* will indicate nonparticipation or refusal, *1* will indicate that there is an agreement in principle to participate but that no formal MOU has been established, and *2* will indicate that a formal MOU has been put into place between the agency and the study team. For the purposes of hypothesis testing, we will aggregate values of *1* and *2*, but the data will be documented in ordinal form so that we can identify whether there is a concern with agencies failing to enter the MOU stage after agreeing in principle to participate.
- Next, the agency recruitment metric *M* for each arm will be the ratio of recruited agencies to total agencies with which active communication has been established: *M*=(*R_R>0_*/*A_A>0_*).

In addition to computation of the primary outcome metric, recruitment activities will be documented in a shared internal database, which will track, at a minimum, the nature, date, and outcome of each instance of correspondence with each community to inform our understanding of the uptake of *PulsePoint-OD* among *PulsePoint* communities and support ongoing mitigation of any issues with recruitment. Using this documentation, we will be able to estimate several secondary descriptive outcomes:

- An estimate of the dose-response uptake of the intervention (eg, mean number and types of correspondence associated with agreement to participate and completion of an MOU)
- An estimate of the average length of time between initial contact and agreement to participate

#### H1: Sample Size, Power, and Analysis

Although we plan for 180 agencies to compose the total sample size, only 120 (66.7%) will be allocated to arms 1 (n=60) and 2 (n=60), meaning that the sample size for this hypothesis is 120. With 60 agencies in each arm, we will have 80% power to detect significant differences (2-tailed α=.05) between arms 1 and 2 in the proportions of agencies that are successfully recruited if the difference in proportions is at least 0.25 (eg, 18/60, 30% recruited in arm 1 vs 33/60, 55% recruited in arm 2). The primary analysis will be conducted using logistic regression, comparing the recruitment rate between the arms. We will report exact P values and commit to a cautious interpretation of the findings, avoiding overreliance on P values as the single arbiter of meaning [64]. We do not expect any missing data in the outcome variable because the nature of its computation is such that missing data mean that either *A*=*0* or *R*=*0* and are therefore built into outcome variable *M*.

The primary study outcome (*M*) will be tested separately for the periods 6 and 12 months after initial recruitment correspondence. Analysis covariates are planned to include, but may not be limited to, community rurality and the community’s percentage vote share for the Republican candidate in the 2020 presidential election (available for 2523 US counties and separable by zip code [65]), given that political orientation was 1 of 2 strongly predictive variables in our regression model examining misperceptions of OEND [39].

### H2: Layperson Responders

#### Overview

Layperson responders who are part of the *PulsePoint* system register with a local first responder agency using their smartphone. Most layperson responders (eg, those who are not also off-duty first responders themselves) are only alerted to incidents in public spaces. No identifying information is collected from these users except the unique ID of the device.

The first responder agency in each community that manages the *PulsePoint* subscription can only determine the number of active users and, broadly, the type of smartphone being used (eg, Android smartphone or iPhone). Each time a first responder agency sends out a push notification, it is provided with an exact count of the number of unique devices, separated by type, that were active in the system and reached by the message.

It is important to note that we will have a count of unique active devices but not necessarily a count of unique active users (eg, it is unlikely but possible that someone will install the app on 2 different mobile phones at the same time). In correspondence with the PulsePoint Foundation, it is likely that the count of unique active devices serves as a very close approximation of unique active users. Furthermore, by virtue of randomization, the distribution of any unmeasured anomalies related to user-device mismatches should be evenly distributed across the arms.

#### Conceptualizing Recruitment for Layperson Responders

Currently, for a *PulsePoint* user to be officially recruited as a CPR responder and to be sent notifications regarding unconscious and unresponsive persons in public places, they need to affirmatively indicate that they are trained in CPR *and* are “willing to assist in case of an emergency.” Individuals who so verify are sent push alerts and auditory alert tones, regardless of whether the app is open, whenever there is an unresponsive and unconscious person within a specific radius of their mobile phone (the radius is specified by the first responder agency for that community) [66].

For the purposes of this study, a layperson responder will be considered to have been recruited for *PulsePoint-OD* if they certify that they have received training on overdose and naloxone and that they currently carry naloxone on their person (both outcomes are of interest, which we describe later in the *Outcome Variables and Measurement* section). Once an MOU is in place with a first responder agency, it will send out a customized baseline push message to all active users containing a link to a landing page. This page will contain brief introductory text explaining the importance of recognizing an overdose when responding to unconscious persons and the efficacy of naloxone, as well as 2 questions asking them to indicate, by pressing radio buttons, whether they have received training on recognizing an overdose and administering naloxone and whether they currently carry naloxone on their person. Additional push messages encouraging users to respond to the certification questions will be sent twice within the month to maximize the number of participants providing baseline data. A user’s responses to these questions will not change their status within the app or the behavior of the app because all users are already alerted to unresponsive or unconscious persons who may have experienced an overdose. This certification procedure will be repeated twice (6 months and 12 months after the baseline certification requests are sent). These messages will emphasize that even users who have previously provided such information should do so again.

The *PulsePoint* app is structured in such a way that push notification hyperlinks can open an external web page in a web browser window inside the app. Using the style guides and templates from the PulsePoint Foundation, the certification web pages will be designed to be similar to the appearance of the app to avoid disrupting the user experience while avoiding cross-contamination of messaging between the study arms. Each web page will be accessible only via the hyperlink in its associated push notification, and users will not be able to navigate among web pages or access the web pages for other study arms.

As multiple data collection reminders will be sent each time (baseline, 6 months, and 12 months), we want to minimize the likelihood of an individual repeatedly certifying their involvement. This risk exists because of the completely anonymous way that individual *PulsePoint* users are managed by the system. Thus, the landing page will be configured to store a cookie [67] on the mobile device. Although cookies are typically perceived as tracking devices, this cookie will not *record anything except whether the user has previously responded to either question on the landing page* during the data collection cycle, and it will be programmed to delete itself after each data collection period (eg, 3 weeks). When users who have already completed the questions return to the landing page, the cookie will prevent the questions from being displayed, although we anticipate that some of the users may clear their smartphone browser cache or actively prevent the use of cookies.

#### Layperson Intervention (Recruitment) Arm 1

**Figure 3:**
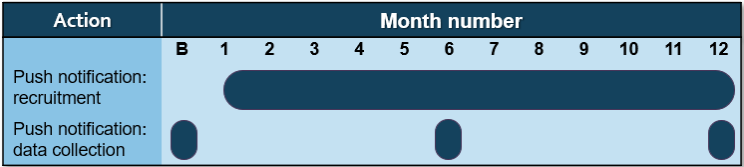
Push notification types and timing relative to agency recruitment. B: baseline.

Users randomized to arm 1 will receive standard monthly recruitment push messages. These push messages will differ from the certification messages because they will contain language intended to foster engagement with OEND services (eg, to get trained and carry naloxone) alongside the link to a landing page (eg, “Are you trained to recognize an overdose? Be a community hero! Click here for more information!”). The messages will be developed based on best practice recruitment principles in cooperation with an external marketing team and then reviewed and finalized by the study team. Messages will be different each month. The monthly time frame was selected based on our need to balance contact with responders and research or expert opinions on push messaging saturation. In a hypothesis-driven objective measure study, increased push messaging frequency was associated with additional app uninstalls *and* lower push message opening rates [68]. This information is generally consistent with the much larger volume of layperson marketing gray literature on push notification optimization (eg, *how-to* blogs [69]). Figure 3 displays the planned timing of the recruitment and data collection messages.

The landing page for recruitment will be updated to include a link to the Community Overdose Responder naloxone training [70], which has been designed specifically for laypersons and integrates elements of OEND programming and *PulsePoint*. Where provided by community first responder agencies, we will also include a list of ways to obtain free or reduced-cost naloxone locally as well as information about upcoming local OEND events. Separately, we note that accessing at-cost naloxone (ie, not free or subsidized) will be relatively easy, in principle, given its transition to OTC status [22].

#### Layperson Intervention (Recruitment) Arm 2

As in the case of the differentiation between agency recruitment in arm 1 and arm 2, the recruitment of layperson responders for arm 2 will follow the same procedures that are described for arm 1 but will use separate messaging. Specifically, the push messages and landing page will contain additional, brief messages that specifically provide factual information that counteracts misperceptions about overdose and naloxone [39]; for example, whereas an example message for arm 1 might be “Are you trained to recognize an overdose? Be a community hero! Click here for more information!” the permutation for arm 2 might be “Are you trained to recognize an overdose? Studies show that most people revived with naloxone will not overdose again within the next year! Click here for more information!”

#### Layperson Intervention (Control) Arm 3

Arm 3 functions as the control arm and will not receive any monthly recruitment messaging or encouragement, but laypersons will still be asked, using push notifications, to provide information at baseline and 6 and 12 months later to document whether they have been trained to recognize an overdose and administer naloxone and whether they currently carry naloxone on their person (eg, “Please let us know whether you have ever been trained to recognize an overdose or if you carry naloxone by clicking here.”). The landing page will likewise contain the questions with radio button responses but will not link to training opportunities or contain additional information.

#### Outcome Variables and Measurement

In H2, we hypothesize that more layperson responders will indicate that they have received OEND programming and carry naloxone on their person within 6 and 12 months of initial contact using procedures in arm 2 (tailored messaging) compared to procedures in arm 1 (standard messaging) and using procedures in either arms 1 or 2 versus arm 3 (control). We will use several different data points to assess our hypothesis.

All push notifications sent out by first responder agencies already return information through the *PulsePoint* app indicating the total number of active devices, by type, that were messaged. As we explained previously, this is a strong proxy measure for the total number of active users messaged because most people use a single smartphone.

For purposes of computation, this total number of recipients of any push notification will be designated as *T*. Next, the total number of individuals who click on the pushed link will be represented by *O*, which will be a number between *0* and *T*. Importantly, the link will also be coded using HTML and CSS so that it sends additional data when clicked, indicating the originating community (cluster identity) and the data collection point (*D*, a value ranging from *0*, which is the initial push message cluster, to *2*, which is the final data collection message set at 12 months after baseline). Subsequently, the number of instances of an individual (ie, unique device) indicating that they have been trained on recognizing an overdose and administering naloxone (represented by *N*, a binary value of *0* or *1*) and whether they currently carry naloxone (represented by *C*, also a binary value of *0* or *1*) will be captured from the landing page itself.

This information will allow us to determine the following, separately by community:

- At baseline, we will calculate the ratio of persons certifying that they are trained (numeric count of *N*) compared to the total number of push message recipients (*T*), the ratio of persons certifying that they carry naloxone (numeric count of *C*) compared to the total number of push message recipients (*T*), and the ratio of individuals who clicked on the link (*O*) compared to the total number of push message recipients (*T*).
- Separately at 6- and 12-month follow-ups, the numbers of certifications of training and carrying naloxone (*N* and *C*, respectively) that were submitted compared to the numbers of push recipients (*T*) in each period (tracked by *D*) and the ratio of individuals who clicked on the link (*O*) each month compared to the number of total push message recipients (*T*).
- Our outcome variables are ratios: *X = (N / T)* for each value of *D* (0, 1, or 2) and *Y = (C / T)* for each value of *D*. The ratio *Z = (O / T)* is a covariate representing engagement with push messages at each value of *D*.

#### Sample Size, Power, and Analysis

Our power analysis for H2 depends on the number of communities that agree to participate during the agency recruitment conducted as part of testing H1. To compare the average proportions of users who certify that they have received overdose education and personally carry naloxone, we sought 80% power to detect significant differences (2-tailed α=.05) among the 3 arms across 3 time points. Assuming a correlation of 0.5 in responses over time, we expect that we will be able to detect a medium effect size ranging from *f*=0.21 (if 50, 83% of the 60 communities participate in each arm on average) to *f*=0.34 (if 20, 33% of the 60 communities participate in each arm on average).

Our primary analyses will be metrics *X* and *Y* (as described previously), which will be analyzed separately. The primary analyses will be conducted using generalized linear mixed models, adjusting the *df* for the number of agencies. As with H1, we will report exact P values and commit to a cautious interpretation of the findings, avoiding overreliance on P values as the single arbiter of meaning [64]. In addition, as in the case of H1, we do not expect any missing data in the outcome variable because the nature of its computation is such that missing data mean that either *N*=*0* or *C*=*0* and are therefore built into outcome variables *X* and *Y*. Study covariates will include but may not be limited to variable *Z* to control for the conversion of push messages to clicks.

### Exploratory Agency Follow-Up

We will conduct a set of intensive follow-up agency interviews with community first responder organizations with whom we have established active communication. We will prioritize qualitative recruitment of organizations and agencies that did not agree to be recruited after establishing correspondence because the elicitation of barriers to participation in the *PulsePoint-OD* program may be most informative when carried out with those who did not participate. This procedure will be similar in principle to, but more extensive than, our brief study of clinical providers who dropped out from a continuing medical education program [71]. However, we will not exclude participating agencies from the qualitative component of the project; instead, we will continue data collection until we have reached a point of theoretical inductive saturation, when new conversations and data no longer seem to add to our understanding of the topic [72]. Interviews will be guided by a semistructured interview guide to be developed during the agency recruitment process and will be coded using the general inductive approach to qualitative analysis [73], which does not make a priori assumptions about the data and allows themes to emerge organically based on repeated readings of the data.

### Ethical Considerations

Ethics approval for the proposed work was determined to fall into 2 separate categories. Agency-level recruitment and interaction were determined not to constitute human participant research (Indiana University Institutional Review Board: 20218). The collection of information from layperson responders was approved under an expedited procedure (Indiana University Institutional Review Board: 20219). Data from layperson responders will be anonymous (no identifiers will be collected). Participation in the intensive follow-up agency interviews will be incentivized with an honorarium (US $500) to the organization to encourage participation.

## Results

At the time of protocol submission, we had not engaged in any data collection or participant recruitment. This research was funded beginning September 14, 2023, by the National Institute on Drug Abuse of the National Institutes of Health (R34DA058162).

## Discussion

### Summary

The primary goal of this study is to understand the feasibility of recruiting current *PulsePoint* communities and layperson responders to complete overdose and naloxone training and to carry naloxone. Our hypotheses are intended to determine whether a specific type of correspondence targeting anticipated misunderstandings around opioid overdose and naloxone performs better when recruiting agencies and laypersons than standard messaging and whether active recruitment of laypersons using either message type performs better than the control.

At the same time, a separate consideration—regardless of the results of null hypothesis significance testing—is an overall sense of recruitment feasibility (eg, data on agency and responder uptake). Successful completion of this study is anticipated to improve the level of naloxone coverage in the United States, which remains insufficient [29]. We are also interested in examining elements that did and did not work and how we would ideally structure a similar recruitment process for a larger, outcomes-focused study (eg, comparing fatal overdose ratios in communities). Our exploratory discussions with agencies will be important to facilitate a better understanding of the mechanisms through which the proposed recruitment procedures did (or did not) work as intended.

Where possible, we have tried to control for anticipated limitations at the stage of study design. Owing to the nature of this real-world study, we still anticipate some limitations in the interpretation of the results. In particular, the degree to which agencies will agree to participate in arm 3 is uncertain because participation does not directly benefit those communities (unlike arms 1 and 2). This would not affect H1 (which only includes arms 1 and 2) but might affect the statistical power of H2. We expect that additional limitations may be uncovered in the process of conducting the study, and we plan to transparently note, and where possible mitigate, all such limitations, providing a comprehensive description of this information in subsequent documentation of the study.

### Conclusions

If we successfully recruit agencies and laypersons and thereby facilitate training around overdose and naloxone and increase the numbers of layperson responders carrying naloxone, the next step will be to conduct a larger study examining opioid-involved overdose reversals using these procedures. This is predicated on current evidence for OEND, which allows us to hypothesize that our successful mobilization of layperson responders might reduce the prevalence of opioid-involved overdose [12-16], a theory that we will then prospectively test.

## Appendix

Multimedia Appendix 1 Peer-review report by the National Institute on Drug Abuse of the National Institutes of Health.

## Abbreviations

CPR: cardiopulmonary resuscitation
MOU: memorandum of understanding
OEND: overdose education and naloxone distribution
OTC: over-the-counter

## Data Availability

Data sharing is not applicable to this paper because no data have been obtained or analyzed at this stage of the study.
