# Supplementary material for: The Feasibility of Using the National PulsePoint Cardiopulmonary Resuscitation Responder Network to Facilitate Overdose Education and Naloxone Distribution: Protocol for a Randomized Controlled Trial
Source: JMIR Res Protoc. 2024 Mar 29;13:e57280. doi: 10.2196/57280 (PMC11015366; doi:10.2196/57280)
Supplement: Multimedia Appendix 1 [file resprot_v13i1e57280_app1.pdf]

1R34DA058162-01A1 AGLEY, JON

RESUME AND SUMMARY OF DISCUSSION: This responsive resubmitted application proposes a 3-arm multi-stage randomized controlled trial (RCT) to assess the feasibility of overdose education and naloxone distribution though an established mobile emergency response app known as PulsePoint. Reviewers found the project highly significant in its potential to inform interventions to swiftly reach those experiencing drug overdose while also reducing stigma associated with naloxone use. If successful, the project could inform cost effective yet broad reaching efforts to reduce overdose. The investigative team is strong with relevant expertise in overdose education and naloxone distribution (OEND), harm reduction, software development, and RCTs. The research environment is excellent with appropriate resources and supports. Strengths of the approach include a rigorous 3-arm RCT design, appropriate statistical analyses, and strong support from the PulsePoint Foundation. Reviewers noted some weaknesses with the approach, including underdevelopment of the qualitative aim, unclear outcomes measures, fidelity measures, and content of messages through PuslePoint. Reviewers had a difference of opinion with respect to the study's conceptual model. Some reviewers found the conceptual model to be clear, while others found the model lacked clarity and characterized it as a mix of a conceptual and logic model. Following discussion, the difference of opinion with respect to the conceptual model was still evident. Findings are expected to have a moderately high to high impact on informing future interventions seeking to reduce community fatal overdose rates.

DESCRIPTION (provided by applicant): For the 12 months ending in September 2022, more than 100,500 Americans died from an overdose, the majority of which involved opioids. Substantial national, state, and local resources have been invested in reducing the prevalence of overdose deaths in the United States. But rather than substantively declining, the annual overdose death count has remained over 100,000 for year-over-year reports since June 2021. There is no obvious, single solution to the overdose epidemic, which is a 'wicked problem' requiring a multifaceted prevention, treatment, and recovery system addressing a wide variety of risk factors. However, it is unequivocally clear that naloxone, an opioid antagonist that can reverse overdose, is a highly effective, rapid response tool that can save lives. What is perhaps less well understood is that naloxone is remarkably underutilized; in 2019, fewer than one quarter of fatal opioid-involved overdoses in the US had any evidence of naloxone administration prior to death. For naloxone to be an effective solution, it must be present at the scene (e.g., a dose must be within a radius of an overdose where it can be used) and someone within that radius also needs to be trained and willing to administer it. Overdose education and naloxone distribution (OEND) programming for laypersons (e.g., bystanders; other citizens who are not first responders or medical professionals) has been shown to be feasible and effective in reducing fatal overdose rates but does not presently have sufficient reach. As a solution, we propose a strategy to leverage the PulsePoint Respond app and network to facilitate OEND programming. The PulsePoint network is an existing, national network of more than 4,400 community first responder agencies who coordinate with 894,744 layperson CPR responders who already have indicated willingness to respond to unconscious and unresponsive persons in public. These layperson responders are notified through the app when a community first response agency deploys an emergency response team to an unconscious or unresponsive person in public who is within a certain radius of the layperson. This means that the infrastructure to deploy individuals to potential opioid overdoses already exists and is active, but the citizen responders have not necessarily been trained (overdose education and naloxone use) or carry naloxone. We will test the feasibility of recruiting PulsePoint agencies and layperson responders for OEND using a 3-arm, multi-stage randomized controlled trial that will assess (1) recruitment of community first responder agencies and (2) layperson engagement with OEND programming across (a) a standard recruitment condition, (b) an overdose/naloxone misinformation debunking condition, and (c) a control condition. We will also conduct qualitative follow-up analyses. Successful completion of this project will directly inform procedures for a follow- up R01 application to

test this approach with an outcomes-focused randomized, controlled trial across a much larger (1,000+) community sample with a goal of meaningfully reducing community fatal overdose rates.

PUBLIC HEALTH RELEVANCE: Despite substantial investment in treatment, prevention, and recovery, the United States continues to experience historically high rates of fatal overdoses, many of which involve opioids. Naloxone, an opioid antagonist, is a highly effective overdose reversal mechanism that can be easily used by laypersons – but it remains remarkably underutilized. This study will test how best to connect a large, existing CPR citizen responder network with opioid education and naloxone distribution programs, with the long-term goal of supporting a national-scale assessment of whether this approach can reduce the prevalence of fatal opioid- involved overdoses.

CRITIQUE 1

Significance: 2
Investigator(s): 1
Innovation: 4
Approach: 3
Environment: 1

Overall Impact: The goal of this proposal is to use an RCT 3 arm design to assess the uptake/feasibility of overdose education and naloxone distribution (OEND) through PulsePoint, an established mobile emergency response application with almost 900,000 active users. They will conduct a feasibility trial with 180 communities randomized to targeted recruitment (via push messages), targeted recruitment plus stigma-focused misinformation debunking, or a control arm. This is a very responsive resubmission, with the investigators eliminating the prior Aim 2, instead focusing on intervention uptake and barriers to participation. These are smart steps to prepare for a subsequent RCT once feasibility has been assessed and improved. Key strengths of the proposed research include using a large extant layperson responder network, well-designed analyses, and appropriate follow-up time points given the focus of the R34 (6 and 12 months). A couple minor-to-moderate weaknesses in the approach slightly dampen my enthusiasm, but overall, this is a very thoughtful and potentially impactful feasibility trial.

1. Significance:

Strengths

- If successful, this project would have broad reach with low levels of additional resource investment given the existence of PulsePoint.
- Naloxone efficacy is dependent on accessing it quickly when needed: Getting it to the right place at the right time. The proposed study is a very clever way to increase the likelihood that an overdose occurs near someone trained, able, and willing to immediately respond.
- Importance of stigma reduction: The study team recently found that there are important misinformed beliefs around naloxone, and identified potential strategies to reduce these stigmatizing beliefs.

Weaknesses

- None noted by reviewer.

2. Investigator(s):

Strengths

- PI Agley is an expert in naloxone access and harm reduction, and has conducted RCTs and feasibility projects. He also has experience with understanding the role of misinformation around naloxone.
- Co-I Henderson has experience recruiting community agencies for OEND, lots of relevant experience.
- The team also includes sufficient expertise in biostatistics, software development, OUDs, and harm reduction.

Weaknesses

- None noted by reviewer.

3. Innovation:

Strengths

- First OEND study to directly address misperceptions of naloxone.
- Leveraging PulsePoint is a novel and efficient approach.

Weaknesses

- Basic (though appropriate) study design, intervention messages.

4. Approach:

Strengths

- Prior work demonstrates their ability to recruit first responder agencies.
- Designed to assess real-world uptake; will have important implications for the eventual RCT. Focus on engagement and recruitment prior to focusing on outcomes will improve the eventual final intervention/study design.
- 3-arm RCT: sufficient power, important differences to explore between all 3 arms.
- Use of “push messages” both for recruitment and data collection is efficient and clever.
- 12-month follow-up appropriate.
- Appropriate statistical analyses, data.

Weaknesses

- Minor: Details about next steps/what the potential RCT will look like are missing.
- Missing detail about the messaging refinement process.
- Will the app include details about where/how to procure naloxone? What about sharing information where/how to procure naloxone not at cost if someone cannot afford it?
- Sample size for qualitative interviews? Will they only include agencies who refused to participate?

5. Environment:

Strengths

- Strong letter of commitment from PulsePoint.
- Indiana University Bloomington and affiliated centers (e.g., Prevention Insights) provide more than sufficient resources necessary to conduct the proposed work.

Weaknesses

- None noted by reviewer.

Study Timeline:

Strengths

- Sufficient time for feasibility trial plus 12-month follow-up.

Weaknesses

- None noted by reviewer.

Protections for Human Subjects:

Acceptable Risks and/or Adequate Protections.

- Exempt; adequate protections given this.

Data and Safety Monitoring Plan (Applicable for Clinical Trials Only):

Not Applicable (No Clinical Trials)

Inclusion Plans:

- Sex/Gender: Distribution justified scientifically.
- Race/Ethnicity:
- Inclusion/Exclusion Based on Age:
- N/A (exemption 4).

Vertebrate Animals:

Not Applicable (No Vertebrate Animals)

Biohazards:

Not Applicable (No Biohazards)

Resubmission:

- Extremely responsive resubmission. Investigators dropped one of the Aims, changed from MPI to single PI, reduced follow-up to 12 (vs. 18) months, better justified and provided more details about the stigma reduction arm, provided more details about material development and refinement, added a conceptual framework.

Resource Sharing Plans:

Not Applicable (No Relevant Resources)

Budget and Period of Support:

Recommend as Requested.

CRITIQUE 2

Significance: 2
Investigator(s): 2
Innovation: 1
Approach: 2
Environment: 1

Overall Impact: This study proposes as 3-arm RCT to test recruitment strategies to enroll agencies and individuals to the PulsePointOD program. Opioid overdoses are a critical public health concern and widespread distribution of naloxone can help prevent many acute overdoses. In addition, misinformation regarding access to naloxone can prevent wide spread uptake. This project will address the need to increase access to naloxone and prevent opioid overdoses. The PI and investigator team have the expertise and experience to carry out the proposed project. This study capitalizes on an existing national infrastructure (PulsePoint) which makes it both innovative and resource expedient. Additional strengths include: use of a 3-arm RCT, intervening on anticipated misinformation can improve uptake of OEND, a clear conceptual framework and a strong letter of support. The revised application was responsive to the previous reviewer critiques. Remaining concerns include: How will this study handle laws that regulate liabilities to bystanders that vary by state and localities? No clear justification for the expected effect size.

1. Significance:

Strengths

- Opioid overdoses are a critical public health concern and widespread distribution of naloxone can help prevent many acute overdoses.
- Misinformation regarding access to naloxone can prevent widespread uptake.

Weaknesses

- Investigators mention that “current approaches to OEND have not succeeded in reaching the threshold of sufficient coverage”. It is unclear what the threshold is and if the proposed approach would reach that threshold.

2. Investigator(s):

Strengths

- The PI and investigator team have the expertise and experience to carry out the proposed project.

Weaknesses

- The biosketch of the project statistician (Dr. Dickinson) is generic. It is unclear what specific statistical methodological expertise they will provide to the project.

3. Innovation:

Strengths

- This study capitalizes on an existing national infrastructure (PulsePoint) which makes it both innovative and resource expedient.

Weaknesses

- None noted by reviewer.

4. Approach:

Strengths

- Use of a 3-arm RCT.
- Intervening on anticipated misinformation can improve uptake of OEND.
- Clear conceptual framework.
- Strong letter of support from the President of PulsePoint Foundation.

Weaknesses

- Laws that regulate liabilities to bystanders may vary by state and localities. How will this be handled in this study?
- No clear justification for the expected effect size (proportional difference of 0.25).

5. Environment:

Strengths

- The institutional environment is adequate to support this project.

Weaknesses

- None noted by reviewer.

Study Timeline:

Strengths

- Appropriate as proposed.

Weaknesses

- None noted by reviewer.

Protections for Human Subjects:

Acceptable Risks and/or Adequate Protections.

Data and Safety Monitoring Plan (Applicable for Clinical Trials Only):

Not Applicable (No Clinical Trials)

Inclusion Plans:

- Sex/Gender: Distribution justified scientifically.
- Race/Ethnicity: Distribution justified scientifically.
- Inclusion/Exclusion Based on Age: Distribution justified scientifically.

Vertebrate Animals:
Not Applicable (No Vertebrate Animals)

Biohazards:
Not Applicable (No Biohazards)

Resubmission:

- This application is responsive to the previous review.

Resource Sharing Plans:
Not Applicable (No Relevant Resources)

Budget and Period of Support:
Recommend as Requested.

CRITIQUE 3

Significance: 2
Investigator(s): 1
Innovation: 2
Approach: 6
Environment: 1

Overall Impact: This R34 application aims to conduct an RCT to get feasibility data on recruitment to facilitate naloxone uptake and use among app users that engage first responders in order to expand reach of the number of trained individuals able to respond to opioid overdose situations. This proposal has high significance and innovation, and a well-trained study team to carry out this work. Weaknesses in the proposal that will result in medium impact mainly stem from the approach. This is resubmission that was responsive to prior critiques however concerns related to the approach remain, including a confusing conceptual model, qualitative methods and analysis missing, one specific aim that may not provide the necessary feasibility data for a subsequent R01 proposal, and lack of fidelity measurement.

1. Significance:

Strengths

- Harm reduction approaches to opioid overdose are currently not reaching their potential to prevent deaths.

- Expanding the number of responders by leveraging existing networks has broad translational importance in naloxone uptake and use.

Weaknesses

- None noted by reviewer.

2. Investigator(s):

Strengths

- Team has extensive experience with opioid overdose. PI is well positioned with relevant experience to lead the study.
- Team brings complimentary expertise in feasibility and evaluation, biostatistics, harm reduction, misinformation, opioid use disorders.

Weaknesses

- None noted by reviewer.

3. Innovation:

Strengths

- Study leverages an existing national emergency response network that will substantially expand reach to harm reduction if the study aims are achieved.
- Expanding expertise to lay health persons for naloxone uptake and use is novel.
- The study will directly address misinformation with naloxone uptake and use.

Weaknesses

- None noted by reviewer.

4. Approach:

Strengths

- Analytic plan for assessing recruitment specific hypotheses is clear.

Weaknesses

- The conceptual model that is now included in this revision is confusing and difficult to follow.
- Qualitative interview procedures and analysis as a subpoint to AIM 1 hypothesis is missing from the application.
- If the subsequent investigation will be a full scale R01, this proposal with only one aim misses opportunities to assess other areas of feasibility.
- Expected outcome of the aims difficult to discern from the proposal.
- No measures of fidelity.

5. Environment:

Strengths

- This is a well-resourced environment with relevant partnerships in place to carry out the proposed study.

Weaknesses

- None noted by reviewer.

Study Timeline:

Strengths

- The timeline is adequate to reach major milestones of the study.

Weaknesses

- None noted by reviewer.

Protections for Human Subjects:

Acceptable Risks and/or Adequate Protections.

- Adequate

Data and Safety Monitoring Plan (Applicable for Clinical Trials Only):

Acceptable

- Adequate

Inclusion Plans:

- Sex/Gender: Distribution justified scientifically.
- Race/Ethnicity: Distribution justified scientifically.
- Inclusion/Exclusion Based on Age: Distribution justified scientifically.
- Adequate

Vertebrate Animals:

Not Applicable (No Vertebrate Animals)

Biohazards:

Not Applicable (No Biohazards)

Resubmission:

- Prior critiques were addressed and moderately strengthened the application.

Resource Sharing Plans:

Acceptable

Budget and Period of Support:

Recommend as Requested.

THE FOLLOWING SECTIONS WERE PREPARED BY THE SCIENTIFIC REVIEW OFFICER TO SUMMARIZE THE OUTCOME OF DISCUSSIONS OF THE REVIEW COMMITTEE, OR REVIEWERS' WRITTEN CRITIQUES, ON THE FOLLOWING ISSUES:

PROTECTION OF HUMAN SUBJECTS: ACCEPTABLE

INCLUSION OF WOMEN PLAN: ACCEPTABLE

INCLUSION OF MINORITIES PLAN: ACCEPTABLE

INCLUSION ACROSS THE LIFESPAN: ACCEPTABLE

COMMITTEE BUDGET RECOMMENDATIONS: The budget was recommended as requested.

Footnotes for 1R34DA058162-01A1; PI Name: Agley, Jon

NIH has modified its policy regarding the receipt of resubmissions (amended applications).See Guide Notice NOT-OD-18-197 at https://grants.nih.gov/grants/guide/notice-files/NOT-OD-18-197.html. The impact/priority score is calculated after discussion of an application by averaging the overall scores (1-9) given by all voting reviewers on the committee and multiplying by 10. The criterion scores are submitted prior to the meeting by the individual reviewers assigned to an application, and are not discussed specifically at the review meeting or calculated into the overall impact score. Some applications also receive a percentile ranking. For details on the review process, see http://grants.nih.gov/grants/peer_review_process.htm#scoring.
